# Application of computerized virtual preoperative planning procedures in comminuted posterior wall acetabular fractures surgery

**DOI:** 10.1186/s13018-022-02937-5

**Published:** 2022-01-29

**Authors:** Yifan Zheng, Jianan Chen, Siyu Yang, Xi Ke, Dan Xu, Guodong Wang, Xianhua Cai, Ximing Liu

**Affiliations:** 1 Department of Orthopedics Surgery, Central Theater General Hospital of Chinese People’s Liberation Army, 627 Wuluo Road, Hongshan District, Wuhan City, Hubei Province China; 2 The First School of Clinical Medicine, Southern Medical University, Guangzhou, Guangdong Province China; 3 Department of Rehabilitation, Central Theater General Hospital of Chinese People’s Liberation Army, 627 Wuluo Road, Hongshan District, Wuhan City, Hubei Province China

**Keywords:** Preoperative planning, Computer-assisted, Acetabular fracture, Posterior wall, Comminuted

## Abstract

**Background:**

The treatment of comminuted posterior wall acetabular fractures remains challenging due to the difficulty in understanding of fracture patterns and lack of appropriate preoperative planning process. Virtual preoperative planning procedures are now being commonly used in orthopedic surgery to aid in management of such complex problems. Our aim was to evaluate the feasibility and clinical value of a new method by applying computerized virtual preoperative planning procedures in the treatment of comminuted posterior wall acetabular fractures.

**Methods:**

A total of 45 patients with comminuted posterior wall acetabular fractures from June 2014 to December 2018 were retrospectively analyzed. Based on the usage of computerized virtual preoperative planning procedures, they were assigned to group A and group B. In group A (24 patients), the new method was applied before surgery. In group B (21 patients), the conventional surgery was performed without assistance of computerized virtual preoperative planning procedures. The two groups were assessed in terms of blood loss, surgical time, reduction quality, fracture healing time, postoperative complications, and hip function.

**Results:**

There were no significant differences in demographic data between the two groups. Patients in group A had significantly less intraoperative blood loss (429.58 vs 570.24 ml, *P* < 0.001) and shorter operation time (154.79 vs 181.90 min, *P* < 0.01) compared to group B. Using the Matta scoring system, the reduction was graded as anatomic in 20 cases, imperfect in three cases and poor in one case in group A, versus 16 cases was graded as anatomic, three as imperfect and two as poor for group B. According to the modified Merle d’Aubigné score, hip function was graded as excellent in 15 cases, good in seven cases, fair in one and poor in one for group A in comparison to 11 cases, seven cases, two cases, and one case for group B, respectively. The reduction quality and hip function did not differ within the two groups (*P* > 0.05). The general postoperative complication rate in group A and group B was 12.5% and 28.6%, respectively, but the difference between the two groups was not statistically significant.

**Conclusion:**

The application of computerized virtual preoperative planning procedures is feasible in comminuted posterior wall acetabular fractures. It helps orthopedic surgeons better understand the fracture characteristics, enables simulation of the reduction process and preoperative planning of internal fixation methods. This new preoperative planning method using a 3D virtual model is a more effective method than conventional method in surgical treatment of comminuted posterior wall acetabular fractures.

*Trial registration* retrospectively registered.

## Background

Posterior wall fracture is the most common type of acetabular fracture, accounting for about 1/4 ~ 1/3 of acetabular fractures [1, 2]. As a fracture of the weight‐bearing joint, restoring the integrity of anatomical structure is of primary importance to ensure the normal contact stress between articular surfaces and to achieve a satisfactory long-term hip function [3, 4]. However, most of the posterior wall fractures are comminuted or have areas of impaction which makes anatomic reduction of the articular surface and the fixation of the fracture very difficult [1, 5]. Several studies have shown that isolated posterior wall fractures or complex acetabular fractures involving the posterior wall are less effective in treatment [1, 6].

When faced with comminuted posterior wall acetabular fractures, appropriate preoperative planning for reduction strategies and internal fixation methods are essential to achieve a good result. The complexity of acetabular anatomy and various types of comminuted posterior wall acetabular fractures make orthopedists difficult to correctly recognize and understand the fracture characteristics, which will influence the sequential surgery planning process. The three-dimensional CT (3D-CT) of pelvis has improved the diagnostic capacity of acetabular fractures [7], but a complete understanding of fracture lines and fragments remains difficult. Orthopedic surgeons cannot make further operations like simulating the reduction procedure and the placement of internal fixations on 3D-CT images either [8].

Currently, with the rapid development of digital orthopedic technology and imaging modalities, a 3D virtual model of the fracture acetabulum can be generated through the medical software, which allows orthopedic surgeons to better understand the fracture patterns, simulate the fracture reduction process and perform virtual preoperative planning of internal fixation [9, 10]. Some studies have reported the application of computer-assisted virtual planning system and obtained good clinical outcomes in complicated acetabular fractures [11, 12]. However, there are still rare studies on the use of computerized virtual preoperative planning procedures for comminuted posterior wall acetabular fractures. Therefore, the purpose of this study was to evaluate the feasibility and clinical value of a new method by applying computerized virtual preoperative planning procedures in the treatment of comminuted posterior wall acetabular fractures.

## Materials and methods

This retrospective case–control study was conducted at the Department of Orthopedics in the General Hospital of Central Theater Command from June 2014 to December 2018. Approval for the study was obtained from the institutional research ethics board. Written informed consent was obtained from all the patients.

### Inclusion and exclusion criteria

The inclusion criteria were: (i) age greater than or equal to 18 years; (ii) isolated posterior acetabular wall fractures with three or more fragments.

The exclusion criteria included: (i) time from injury to surgery over 3 weeks; (ii) open or pathologic posterior wall acetabular fractures; (iii) abnormal activity of the hip joint before injury; (iv) complex acetabular fracture types, concomitant femoral head fracture, or pelvic fracture; (v) the follow-up period was less than 12 months and had incomplete radiographic data.

### Patient demographics and characteristics

According to the inclusion and exclusion criteria above, 45 patients with comminuted posterior wall acetabular fractures admitted into our department from June 2014 to December 2018 were enrolled in this study. The patients were divided into two groups according to whether the surgery was performed using the new method of computerized virtual preoperative planning procedures (Group A) or the conventional method (Group B). The new method included reconstruction of a 3D virtual fracture model on medical software, virtual fracture reduction, and planning of internal fixation. Group A consisted of 24 patients. Group B comprised 21 patients who were treated by the conventional technique. The two groups had comparable baseline characteristics, including age, gender distribution, mechanism of injury, fracture side, concomitant injuries, hip dislocation, preoperative sciatic nerve damage, and time to surgery (Table 1).

**Table 1:** The baseline characters of patients

Variables	Group A	Group B	Test value	*P* value
Number of patients	24	21		
Age (years)	46.79 ± 11.28	44.38 ± 11.18	*t* = 0.718	0.477
Gender				
Male	18	16	*χ*^2^ = 0.009	0.926
Female	6	5		
Mechanism of injury				
Fall from height	5	4	*χ*^2^ = 0.045	0.978
Traffic accident	16	14		
Other injuries	3	3		
Fracture side				
Right	15	13	*χ*^2^ = 0.002	0.967
Left	9	8		
Concomitant injuries				
Yes	10	7	*χ*^2^ = 0.331	0.565
No	14	14		
Hip dislocation	17	15	*χ*^2^ = 0.002	0.965
Preoperative sciatic nerve damage	5	3	*χ*^2^ = 0.033	0.855
Time to surgery (days)	8.88 ± 3.53	9.19 ± 3.57	*t* = − 0.297	0.768

Concomitant injuries include brain injuries, chest injuries, abdomen injuries, spine fracture, and limb fracture

### Preoperative preparation

Anteroposterior (AP) view and two oblique pelvic radiographs (Judet views) were taken and used to primarily determine fracture type. A 3D-CT scan of the pelvis was used to diagnose more specific injury characteristics (loose intra-articular fragments, femoral head lesions, and marginal impaction). The hip dislocation was managed by closed reduction under general anesthesia at the emergency department within 12 h from injury, and femoral or tibial skeletal traction was applied after reduction while awaiting surgery.

### Group A

**Fig. 1:**
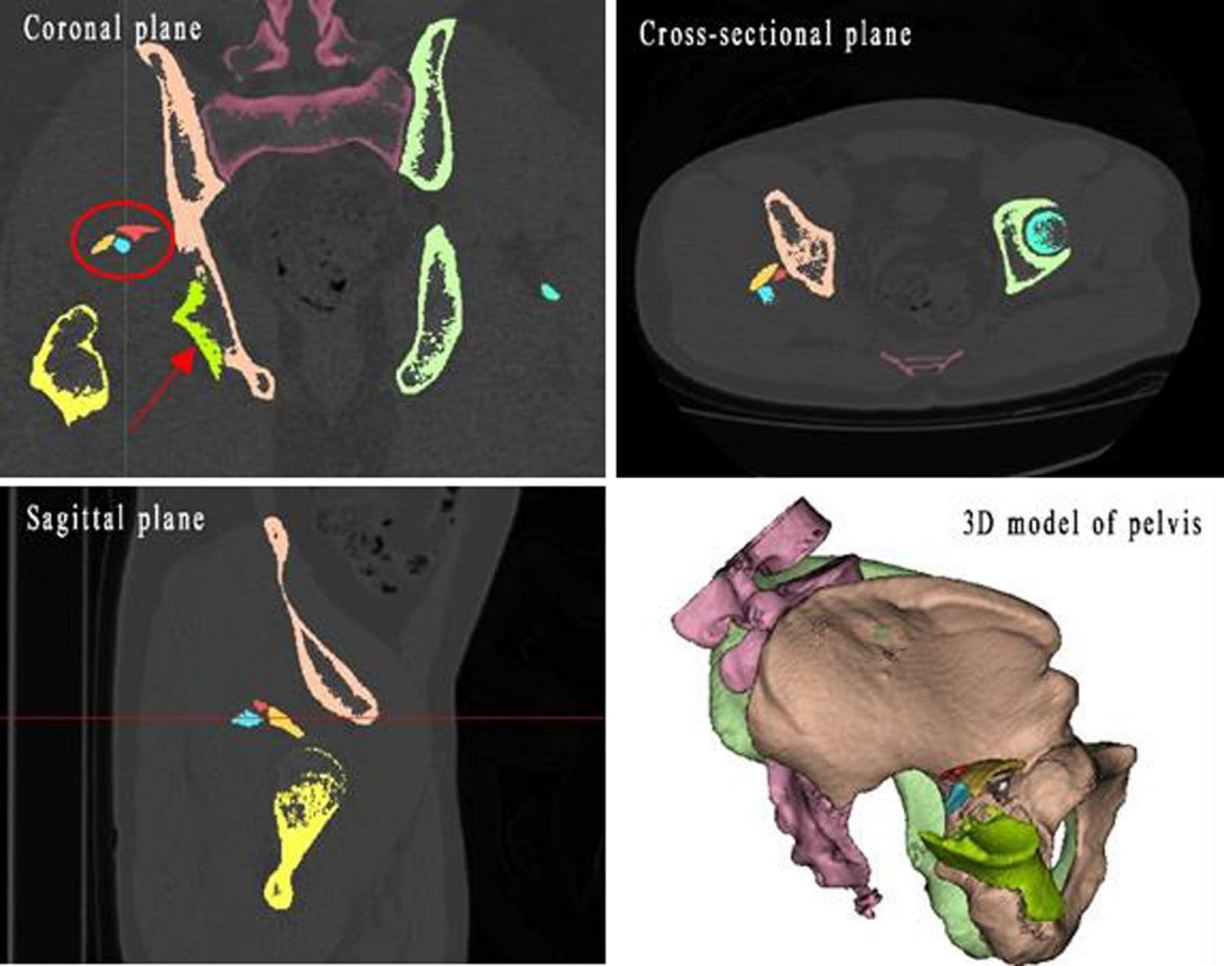
Each fragment was segmented manually in all slices in all three planes then was given to different colors (the blue, red, yellow, and green represent four individual fragments in the right acetabulum. Marked by a red circle and a red arrow). The 3D virtual model of the pelvis possessed separate fragments was reconstructed and could be moved freely by the users

**Fig. 2:**
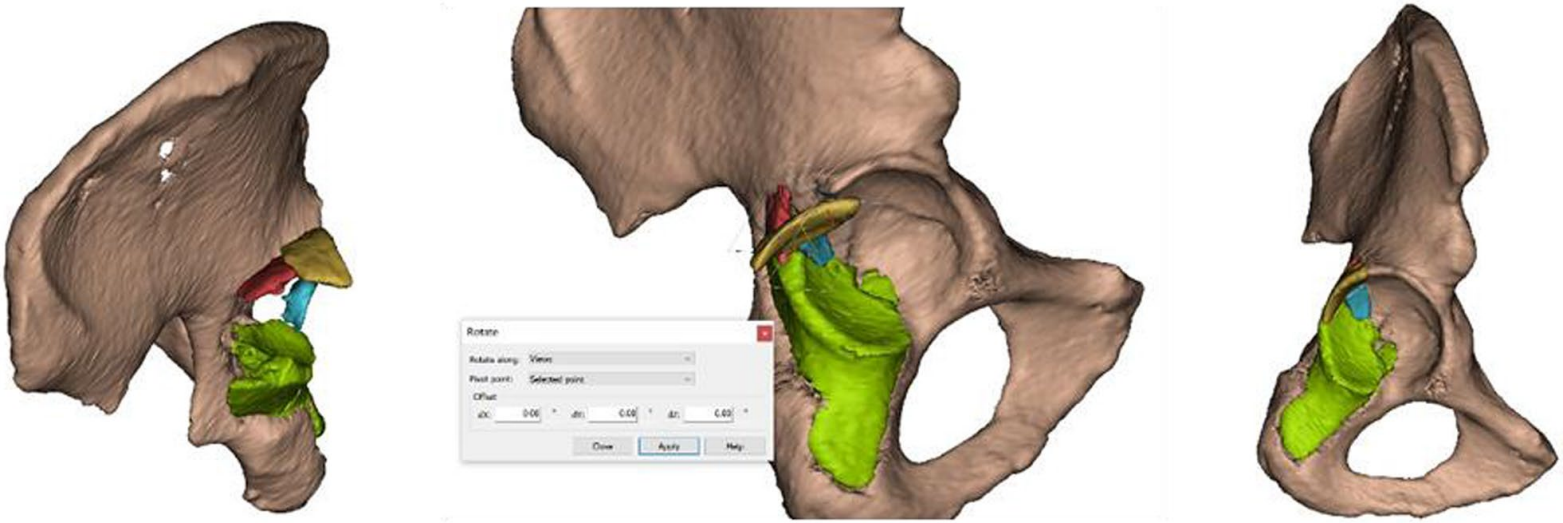
Bone fragments were moved and rotated in all three planes to achieve a satisfactory reduction

**Fig. 3:**
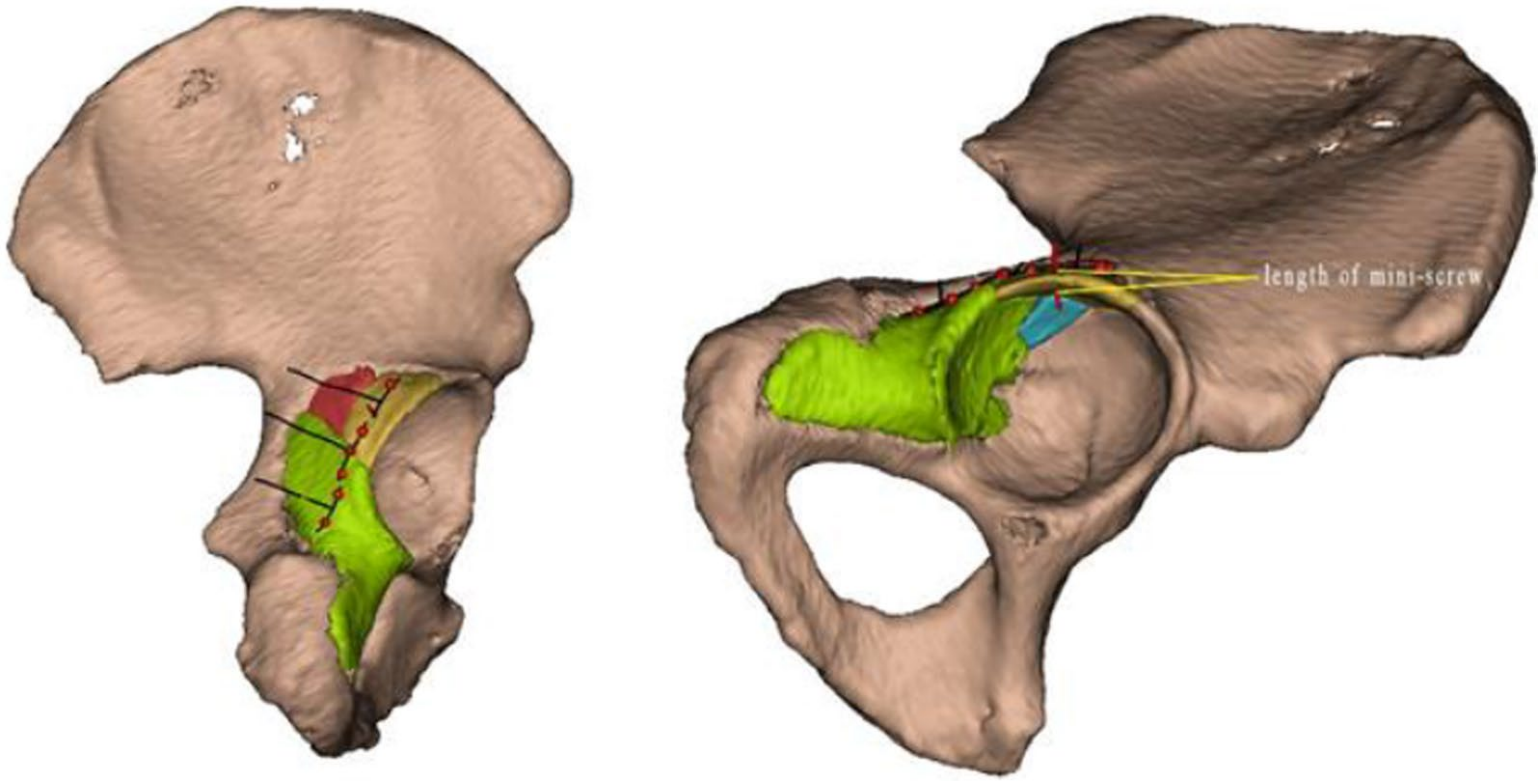
The position,number and type of miniplates (marked by black lines) could be determined according to the fragments’ distribution on the post-reduction model. The red points show fixing points of mini-screws, and the length of mini-screws which were placed perpendicularly to the bone surface could be measured (the red line represents the direction of the mini-screw)

Computerized virtual preoperative planning procedures: The data of CT scan of the pelvis (volume thickness, 1 mm; 64-detector, Siemens AG, Germany) were exported to the Digital Imaging and Communications in Medicine (DICOM) file and imported into Materialize’s interactive medical image control system (Mimics) 20.0 software (Materialize, Belgium) on a personal computer. The reconstruction process of a 3D virtual pelvic model on software was as follows: firstly, we should select a suitable mask restricted to the bone; secondly, we needed to edit the mask manually in all slices in all three planes in order to make individual fragments separated. After that, different colors were assigned to different fracture fragments; lastly, a 3D virtual pelvic model that possessed independent fragments was reconstructed for virtual preoperative planning (Fig. 1). During the virtual preoperative planning procedures, the 3D virtual pelvic model could be turned in all directions so that surgeons could better observe the fracture nature and found more or key problems that needed attention during the real surgery. The femoral head and bone fragments could be removed to observe spatial relationships. Then bone fragments were moved and rotated in all three planes to achieve a satisfactory reduction (Fig. 2). At last, the position, number and type of miniplates (miniplates refer to metacarpal and phalangeal plates, which are used for comminuted posterior wall acetabular fractures in our trauma center) could be determined according to the fragments’ distribution on the post-reduction model. As the mini-screw was placed perpendicularly to the bone surface in most cases, its length could also be measured in Mimics software, especially for fixation of marginal fragments (Fig. 3).

### Surgical technique

**Fig. 4:**
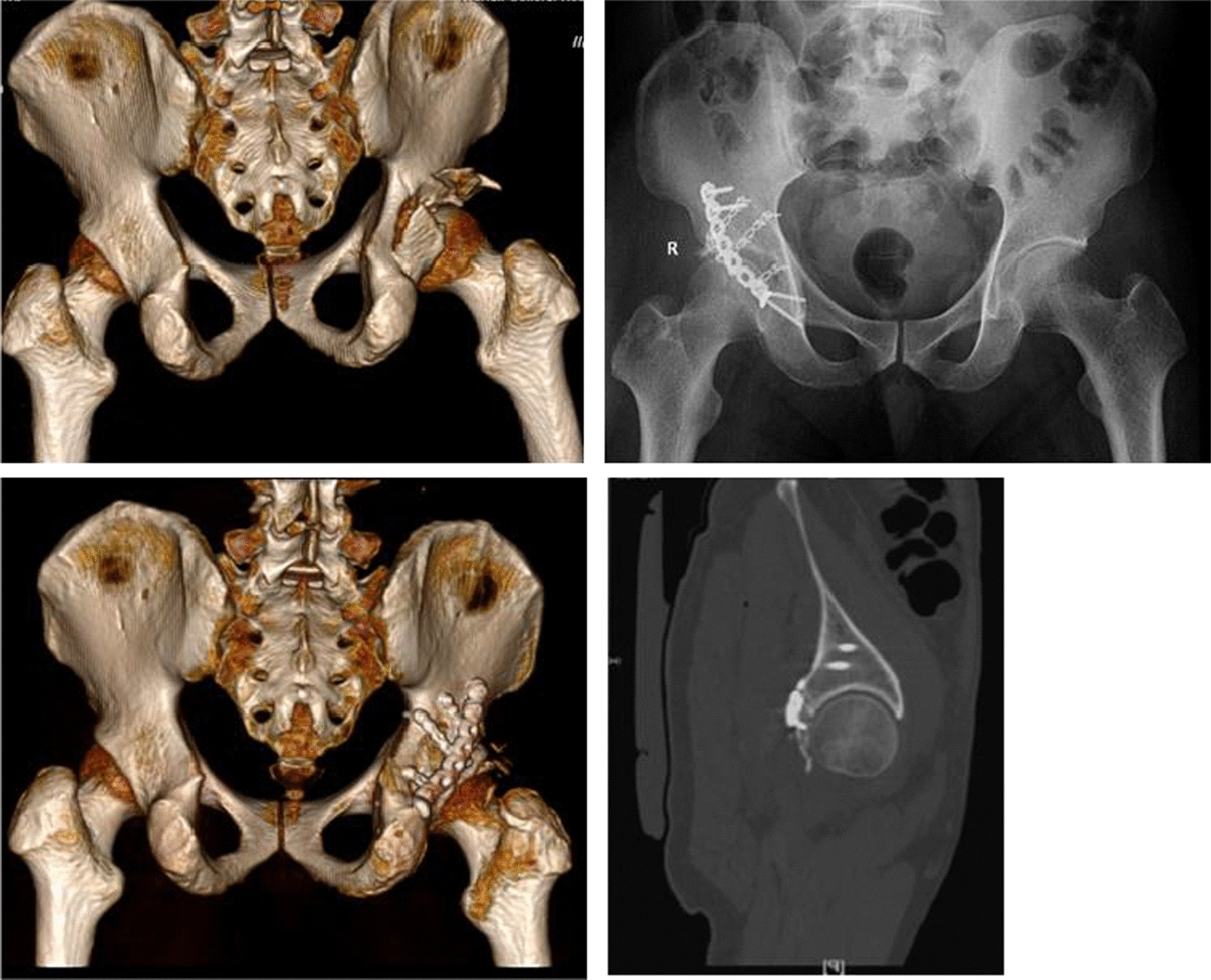
The final surgical fixation method was similar to the virtual preoperative planning results above. The mini-screw was in a good position

All the procedures were performed with the patient under general anesthesia and were positioned laterally on a radiolucent table. All surgeries were performed by two senior surgeons. The posterior wall was exposed using the standard Kocher-Langenbeck approach. In all cases, the sciatic nerve was firstly identified and then protected. The soft tissue and capsule attached to the fragments were preserved. With traction of the affected lower limb, the small loose fragments and hematoma inside the joint were carefully explored and cleared. The femoral head was used as a template to reduce the posterior wall fracture. Once the marginal impaction of the articular surface was present, the impaction was elevated, and cancellous bone from the iliac was grafted to fill the defect. With detailed preoperative planning, try to anatomically reduce each fragment and place the miniplate according to virtual preoperative planning results. Once the miniplate was placed close to the acetabular rim, the length of mini-screw previously measured in the 3D virtual model was used as a reference. Finally, the reconstruction plate was appropriately contoured to accommodate the shape of the posterior wall then placed over the miniplate to increase its stability and prevent postoperative loss of fixation. Intraoperative fluoroscopy was performed to confirm the fracture reduction and hardware position (Fig. 4).

### Group B

Conventionally, surgeons learned about the fracture and made the surgical plan through 3D-CT of the pelvis. The same Kocher–Langenbeck approach was adopted in every case. Miniplates and the reconstruction plate were used in treatment without assistance of virtual preoperative planning procedures.

### Postoperative management

Both groups received the same postoperative management. Prophylactic antibiotic (cefazolin sodium) was given postoperatively and was discontinued 48 h after surgery when the drainage tube was removed. All patients routinely received low-molecular-weight heparin as an anticoagulant therapy. No prophylaxis agent for heterotopic ossification was used. Rehabilitation exercises after surgery were directed by our rehabilitation doctors. Patients received radiological examinations within one-week post-operation, including three standard pelvic plain films (AP view and Judet views) and 3D-CT of pelvis. Patients received routine follow-up at 1, 2, 3, 6, and 12 months postoperatively and annually thereafter. Fracture union, clinical function, and complications were recorded during the follow-up visit.

### Assessment parameters

Surgery-related data were recorded, including blood loss, operation time, and reduction of fracture. Perioperative and postoperative complications were also recorded. Matta’s criteria [13] were used to evaluate the fracture reduction quality based on residual displacement in the three standard pelvic plain films: anatomic (< 1 mm), imperfect (2–3 mm), and poor (> 3 mm). At the final follow-up, the modified Merle d’Aubigné scoring system [14] was used to assess the hip function, the clinical outcomes were graded as excellent (18 points), good (15–17 points), fair (13 or 14 points), or poor (< 13 points).

### Statistical analysis

Statistical analysis was provided by SPSS software version 20.0 (SPSS Inc, Chicago, Illinois). Continuous variables with normal distribution were presented as mean ± standard deviations. Two-group comparisons were performed using a t-test for independent samples. Categorical variables were presented as absolute (n) and relative frequencies (%). The count data were analyzed by *χ*^2^ test, and the rank data were analyzed using the Wilcoxon rank sum test. *P* value < 0.05 indicated a statistically significant difference.

## Results

### Follow up

Patients were followed up in our outpatient clinics. The mean follow-up time of group A and group B was 26.83 ± 9.12 months and 28.95 ± 8.59 months, respectively (*P* = 0.429). The fracture healing time was not different between group A and group B (16.13 ± 2.23 vs. 16.38 ± 2.13 weeks, *P* = 0.697). There was no evidence of intraarticular screw placement, loosening, and migration of the miniplates. No perioperative complications such as iatrogenic sciatic nerve injury, deep venous thrombus, and wound infection occurred in both groups. Patients with sciatic nerve injury recovered completely within six months after symptomatic treatment of neurotrophic drugs.

### Surgical details

The blood loss in group A was less than that in group B. The difference was statistically significant (*P* < 0.001). Surgical time in group A was also found to be significantly shorter than that in group B (*P* = 0.002). According to the Matta scoring system, the quality of reduction was graded as anatomic in 20 (83.3%) cases, imperfect in three (12.5%) cases and poor in one (4.2%) case in group A. In group B, the quality of reduction was graded as anatomic in 16 (76.2%) cases, imperfect in three (14.3%) and poor in two (9.5%). There was no significant statistical difference in fracture reduction between the two groups (*P* = 0.524) (Table 2).

**Table 2:** The surgical outcomes

Variables	Group A (*n* = 24)	Group B (*n* = 21)	Test value	*P* value
Blood loss (ml)	429.58 ± 101.28	570.24 ± 120.20	*t* = − 4.26	0.000
Surgical time (min)	154.79 ± 23.93	181.90 ± 30.88	*t* = − 3.31	0.002
Quality of reduction				
Anatomic	20 (83.3%)	16 (76.2%)	z = − 0.637	0.524
Imperfect	3 (12.5%)	3 (14.3%)		
Poor	1 (4.2%)	2 (9.5%)		

### Scoring of hip function

**Fig. 5:**
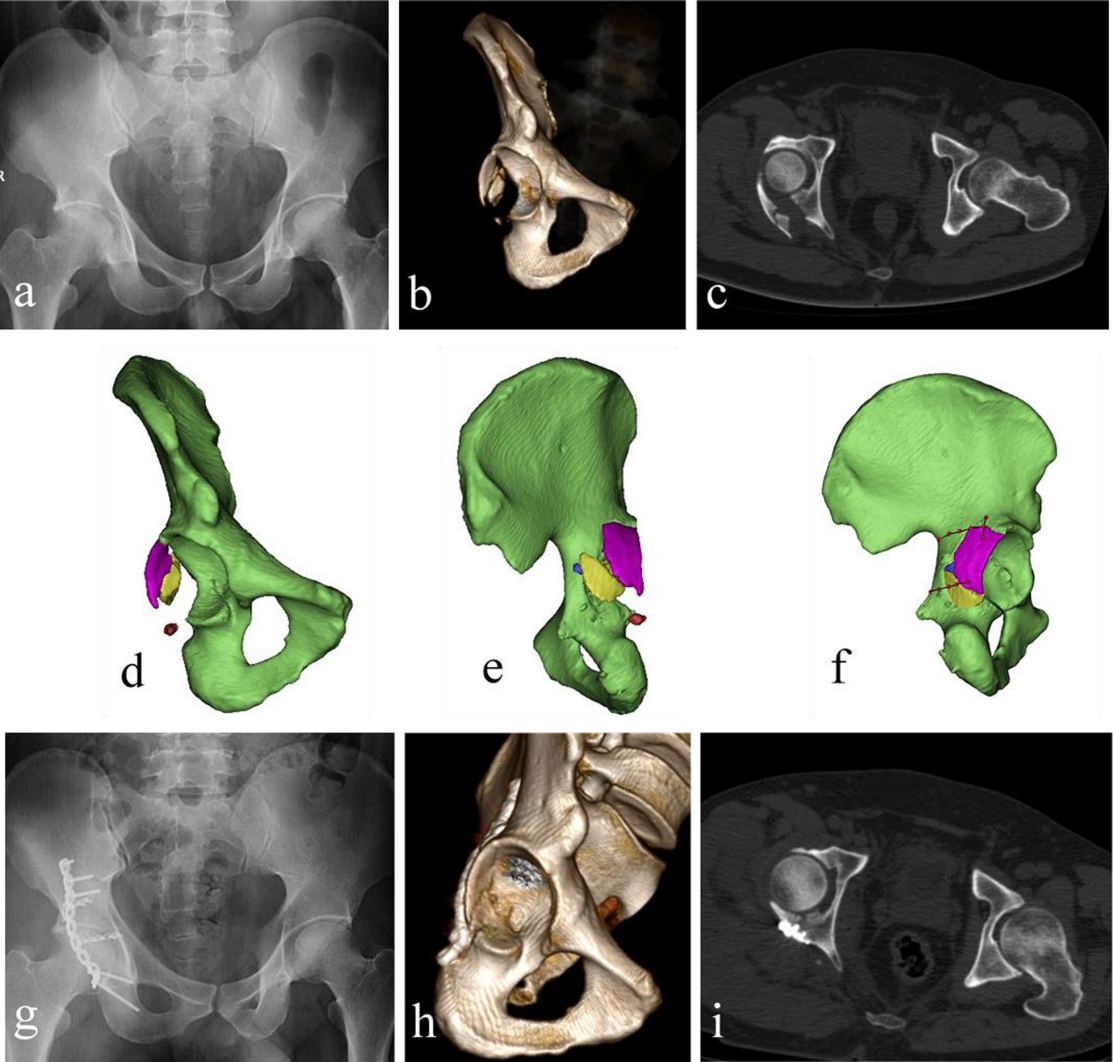
A typical case: A 40-year-old man presented with comminuted posterior wall acetabular fractures of the right acetabulum following a traffic accident. The computerized virtual preoperative planning procedures were applied for the treatment. Preoperative AP view (**a**), 3D-CT (**b**), and cross-sectional CT image (**c**) show comminuted posterior wall acetabular fractures with significant displacement. A 3D virtual model of right acetabulum with separate fragments was reconstructed (**d**, **e**). Preoperative planning of internal fixation methods was achieved on the post-reduction model (**f**). Postoperative AP view (**g**), 3D-CT (**h**), and cross-sectional CT image (**i**) show an anatomical reduction according to Matta grading score

According to the modified Merle d’Aubigné score, the function outcomes at the final follow up in group A were graded as excellent in 15 (62.5%) patients, good in seven (29.1%), fair in one (4.2%), and one (4.2%) in poor which were similar to those in group B (excellent in 11 (52.4%), good in seven (33.3%), fair in two (9.5%), and poor in one (4.8%) (*P* = 0.462) (Table 3). One typical case is shown in Fig. 5.

**Table 3:** Clinical outcomes according to the modified Merle d’Aubigné score

Group	Excellent	Good	Fair	Poor	Test value	*P* value
Group A (*n* = 24)	15 (62.5%)	7 (29.1%)	1 (4.2%)	1 (4.2%)	z = − 0.736	0.462
Group B (*n* = 21)	11 (52.4%)	7 (33.3%)	2 (9.5%)	1 (4.8%)

### Postoperative complications

The posttraumatic arthritis was identified in two (8.3%) cases in group A and three (14.3%) cases in group B. In each of the two groups, there was one patient who had serious symptoms and underwent total hip arthroplasty. No avascular necrosis of femoral head was observed in group A, but one in group B was found hip subluxation three months after surgery, X-ray showed the collapse of femoral head six months later. Due to poor function, a total hip arthroplasty was performed one-year post-operation. One (4.2%) patient developed heterotopic ossification (HO) in group A and was graded as class I according to the classification system by Brooker et al. [15]. Two (9.5%) patients developed HO (one of class I and one of class II) in group B. None HO had contributed negatively to hip function in both groups (Table 4).

**Table 4:** Postoperative complications at the final follow-up

Complications	Group A (*n* = 24)	Group B (*n* = 21)	Test value	*P* value
Posttraumatic arthritis	2 (8.3%)	3 (14.3%)		
Avascular necrosis of femoral head	0	1 (4.8%)		
Heterotopic ossification	1 (4.2%)	2 (9.5%)		
Incidence of complication (*n*, %)	3 (12.5%)	6 (28.6%)	*χ*^2^ = 1.808	0.179

## Discussion

Acetabular fractures are not common yet they are usually caused by high energy accidents. The posterior wall is the most susceptible part, and the majority of fractures are severe [1, 5]. Surgical treatment aims to achieve anatomic reduction, restore articular congruity, and early functional exercise for a satisfactory hip function. However, the surgical treatment of posterior wall acetabular fractures is challenging. It is not only because of the deep and complex osseous geometry of the acetabulum and nearby numerous vascular and nervous elements, but also due to the variety of the comminuted fracture patterns, and the limited view and fixation methods for open reduction and internal fixation. Matta [13] reported that 22 patients who suffered posterior wall fracture in his retrospective study, only 15 (68%) obtained a good-to-excellent clinical outcome, with a poor result in the remaining seven (32%). Saterbak et al. [16] reported that seven cases in their study had complete loss of joint space within one year after surgery, accounting for 35% of a total of 20 posterior wall fractures, and comminution of fractures was found to worsen the clinical result. Although some new fixation methods have been introduced to improve treatment for comminuted posterior wall acetabular fractures [17–19], they are still in the preliminary application stage and have not been popularized in clinical practice. The outcomes of acetabular fractures depend largely upon the quality of the articular reduction [4, 6, 20], the non-sufficient understanding of the fracture patterns and the inadequate surgical planning will adversely affect surgical outcomes, especially for young inexperienced surgeons. Therefore, a new technique is required to facilitate the comprehension of the full extent of fracture and planning of the surgery.

In recent years, advances in image processing and computer technology have permitted the virtual preoperative planning of orthopedic procedures, which have been explored and applied in the treatment of acetabular fractures, and it shows a good prospect [9, 21]. The computerized virtual preoperative planning procedures applied in this study consists of three consecutive steps, including the reconstruction of a 3D virtual model, the virtual fracture reduction, and the planning of internal fixation methods. The plan was based on visualization of a 3D model of the pelvis, orthopedic surgeons were able to fully understand the characteristics and the spatial relationship of the fragments through freely removing the femoral head or bone fragments. We could better manage the fracture fragments and design the optimal reduction sequential steps to perform high-quality internal fixation under the assistance of virtual preoperative planning procedures. Citak et al. [8] compared the 3D virtual planning method for acetabular fractures with the traditional 2D planning method and found that the former method could increase the accuracy of reduction and reduce the time of fracture reduction via pelvis model trials. Hu et al. [9] applied virtual surgical procedure for acetabular fractures and compared it with real surgery with respect to operative approach, plate length, and screw count. They found an agreement between virtual surgical plan and real surgery in all patients. Their results demonstrated that the virtual surgical procedure for acetabular fractures is feasible and useful clinically for surgeons to determine surgical planning.

In this study, we compared the results of comminuted posterior wall acetabular fractures treated with new preoperative preparation method using a 3D virtual model (Group A) versus the conventional method (Group B). We found that the use of computerized virtual preoperative planning procedures led to better surgical outcomes, both intraoperative blood loss and surgical time were reduced significantly by using this new technology. Key surgical cognitive skills are mental readiness, flexible decision-making, forward planning, and awareness of potential problems. Computerized virtual preoperative planning procedures in acetabular fractures allow to develop these skills for a better surgical process [10]. In the conventional surgery, surgeons evaluate the injury pattern by utilizing plain radiographs and CT scan with 3D reconstructions and determine the fracture reduction plans and internal fixation methods intraoperatively, therefore surgical skill and experience are important factors in determining a successful outcome. In that case, it has disadvantages of longer operative time, more blood loss, worse reduction quality, and risk of screws penetrated joint cavity [4, 22]. When the new method of virtual preoperative planning was performed, we could avoid extensive dissection and soft tissue stripping at the time of surgery, repeated manipulations of fracture reduction and adjustments of internal fixation, which led to decrease the operative time and blood loss. We also measured the mini-screw length in the 3D virtual model, especially for fixation points in dangerous areas, which enhanced the safety when placing screws. Our results also showed that we were able to achieve higher fracture reduction quality and hip-function scores with virtual preoperative planning procedures in comminuted posterior wall acetabular fractures, although the differences were not significant.

Our study had several limitations. First, virtual placement of internal fixation was not performed in the study, and only used lines to mark the positions of miniplates and points to represent the mini-screws’ fixation positions. However, since the miniplate does not have a high demand for contouring, and the mini-screws are routinely placed perpendicularly to the bone surface. So, it does not have obvious influences on real surgery. Second, in the current computerized virtual preoperative planning procedures, the segmentation between different bone fragments depends on manual segmentation, which entails considerable use of time. Especially in comminuted fractures, the fragments are relatively more and smaller so that the segmentation of different fragments may not be realized in some cases.

## Conclusion

The application of computerized virtual preoperative planning procedures is feasible in comminuted posterior wall acetabular fractures. It helps orthopedic surgeons better understand the fracture characteristics, enables simulation of the reduction process and preoperative planning of internal fixation methods. This new preoperative planning method using a 3D virtual model is a more effective method than conventional method in surgical treatment of comminuted posterior wall acetabular fractures.

## Data Availability

The datasets used or analyzed during the current study are available from the corresponding author on reasonable request.

## Abbreviations

3D: Three-dimensional
HO: Heterotopic ossification
DICOM: Digital Imaging and Communications in Medicine
